# Characterization of the internal quality of turkey eggs according to their commercial grading

**DOI:** 10.1016/j.vas.2025.100470

**Published:** 2025-06-17

**Authors:** José Ignacio Salgado Pardo, Antonio González Ariza, José Manuel León Jurado, Juan Vicente Delgado Bermejo, María Esperanza Camacho Vallejo

**Affiliations:** a Department of Genetics, Faculty of Veterinary Sciences, University of Córdoba, Córdoba 14071, Spain; b Agropecuary Provincial Centre, Diputación de Córdoba, Córdoba 14071, Spain; c Andalusian Institute of Agricultural and Fisheries Research and Training (IFAPA), Alameda del Obispo 14004, Córdoba, Spain

**Keywords:** Albumen quality, *Meleagris gallopavo*, Egg weight, Shape index, Yolk diameter

## Abstract

The present study aimed to determine the influence of egg weight and shape index on the internal quality of turkey eggs. To this aim, a total of 197 turkey eggs were measured including external and internal egg quality traits. Different egg categories were built attending to the weight terciles of the sample and the commercial shape index standards. A Discriminant Canonical Analysis (DCA) was performed using these categories as dependent variables, while the internal quality attributes acted as explanatory variables. The yolk percentage was the only variable reporting multicollinearity and was therefore removed from further analysis. The Pillai’s trace criterion was significative (*p* < 0.05) and validated the performance of the DCA. Moreover, the cross-validation test reported a high accuracy of correct assignments (95.88 %), which validated the applicability of the statistic model. The diameter and lightness of the yolk, the proportion of shell in the overall weight, and the height and pH of the albumen were the variables reporting discriminatory ability across weight and shape index groups. Similar associations between the internal quality and egg weight were found in the literature, while the connections with the shape index were less frequent. The present work eases the efficient determination of increased-quality eggs from their external appearance. However, quality perception varies with the consumer preferences of each market. Therefore, heavier eggs will offer larger yolks and reduced shell percentage in weight, while lighter and rounder eggs will tend to present darker yolks, with taller albumen and lower gas exchange during storage.

## Introduction

**Fig. 1:**
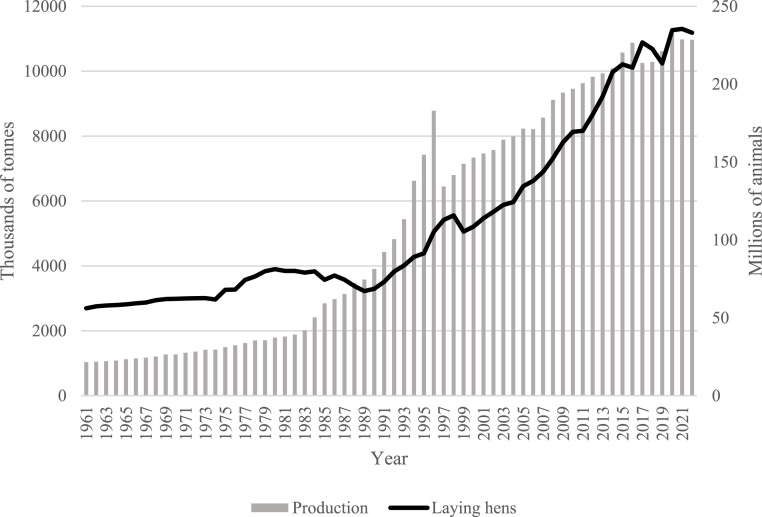
Annual evolution of egg production and number of hens from poultry other than chicken (data source: FAOSTAT database).

The most widespread poultry species are chicken, duck, quails, turkeys, pigeons, guinea fowl, and geese (Istiak and Khaliduzzaman, 2022). This wide group of birds contributes to the supply of human diet worldwide, in which eggs stand as the cheapest among the different animal protein sources (Istiak and Khaliduzzaman, 2022). Intended to build up a mature chick at hatching, eggs contain high-value nutrients to the human diet as essential amino acids and unsaturated fatty acids, iron, phosphate, and fat-soluble vitamins (Istiak and Khaliduzzaman, 2022; Kokoszyński, 2017; Sharif et al., 2018). Worldwide egg consumption increases at a rate of approximately 2 % annually (Sharif et al., 2018), which is almost completely covered by commercial chickens (Abadula et al., 2022; Bondoc et al., 2020; Istiak and Khaliduzzaman, 2022; Kokoszyński, 2017; Tserveni-Goussi and Fortomaris, 2011). However, other poultry species constitute an important source of edible eggs as well (Bondoc et al., 2020; Mia, 2020; Miah et al., 2020; Safiyu et al., 2019; Wong et al., 2017). Egg production in poultry species different to chicken has also increased during the last years according to the FAO database (FAO, 2025). The number of layers and egg production of these birds has doubled and tripled respectively over the last three decades, as shown in Fig. 1.

These ‘alternative’ poultry species are most commonly found in the backyard and grazing systems, commonly known as ‘village poultry’ in developing countries (Abadula et al., 2022; Mia, 2020). They support household food security and ameliorate poverty status in marginal communities (Abadula et al., 2022; Istiak and Khaliduzzaman, 2022; Mia, 2020; Safiyu et al., 2019; Wong et al., 2017) which, according to FAO, comprised about 736 million people in 2015 (10 % of the world’s population) (FAO, 2019). On the other hand, eggs from these species have increased in popularity in urbanized regions (Kokoszyński, 2017; Tserveni-Goussi and Fortomaris, 2011), where they are occasionally found as specialist products sold in exclusive restaurants or stores (Belyavin, 2003). In this line, turkey is a cosmopolitan species found worldwide. Due to their great climate adaptation and scavenging aptitude (Mia, 2020; Safiyu et al., 2019), turkeys have reached backyard productions in almost every part of the world (Mia, 2020; Miah et al., 2020; Safiyu et al., 2019). In addition, their excellent meat quality and high carcass yield have meant that turkeys have found a market alongside chickens in the commercial industry (Mia, 2020; Miah et al., 2020; Safiyu et al., 2019). However, its short laying season and high nesting behavior make the turkey a less productive poultry species in terms of egg laying (Bondoc et al., 2020; Kokoszyński, 2017; Tserveni-Goussi and Fortomaris, 2011). As a result, the commercial turkey industry has mostly allocated eggs for hatching rather than human consumption, whereby egg marketing strategies and distribution channels are still undeveloped (Kokoszyński, 2017; Portillo-Salgado et al., 2020; Tserveni-Goussi and Fortomaris, 2011). This may explain why the consumption of turkey eggs mainly occurs in rural areas, as self-consumption or short trade chains (Akinola and Essien, 2011; Portillo-Salgado et al., 2020; Safiyu et al., 2019; Yassin et al., 2013).

Egg quality involves all parameters influencing consumer acceptance as cleanliness, freshness, size, shell quality, and yolk index (Isidahomen et al., 2014). Moreover, the study of egg quality is also applicable to breeding traits such as hatchability and health during the early life of the chick (Abacı et al., 2023; González Ariza et al., 2022b; Isidahomen et al., 2014). Physical egg quality research is generally divided into internal and external quality (Abacı et al., 2023), attending to the need to break the egg to measure traits (González Ariza et al., 2022a). Despite their results being highly accurate, destructive methods are time-consuming and, since eggs measured cannot be consumed, only a portion of eggs are commonly sampled (Istiak and Khaliduzzaman, 2022). In this respect, external, non-destructive methods have been developed involving spectroscopic, computer vision, electronic nose, fluorescence, and terahertz waves (Aboonajmi and Mostafaei, 2022). However, these methods are costly, difficult to employ, and can only be carried out in laboratories.

For this reason, a cheap and non-destructive approach to egg quality should be developed to ease the study of egg quality when scarce resources are available. In this line, egg weight and shape index have been described as predictors of their physical internal quality (Portillo-Salgado et al., 2020). Egg weight is one of the main phenotypic quality traits studied in poultry and is known to be highly associated with the albumen and yolk weight, as well as with the shell’s quality (Bondoc et al., 2020; González Ariza et al., 2022a; Isidahomen et al., 2014; Kul and Seker, 2004; Özçelik, 2002; Şekeroǧlu and Altuntaş, 2009; Sezai et al., 2013; Shi et al., 2009). On the other hand, shape index has also been associated with shell quality and albumen yield (Duman et al., 2016; Sekeroglu et al., 2000; Sezai et al., 2013). In this respect, research on the correlation between internal and external quality traits has mainly been carried out on chicken and quail eggs (Abacı et al., 2023; Duman et al., 2016; Kul and Seker, 2004; Özçelik, 2002), while there is scant literature on the overall quality of turkey eggs (Portillo-Salgado et al., 2020; Sezai et al., 2013; Sun et al., 2019). In addition, the research approaching the association between eggs’ internal and external quality in the species is even more scarce, and isolated papers addressing different breeds and environments are found. Some have focused on the effect of external traits on hatchability (Andrews, 1972; Hristakieva et al., 2017; Oblakova, 2006), while those addressing organoleptic traits have shown extended storages until measures (Portillo-Salgado et al., 2020) or a short collection period (Yahaya et al., 2021).

Therefore, the present article aims to study the combined effect of egg weight and shape index on physical internal quality parameters through a DCA. Identifying the most explanatory quality traits would lead to a better understanding of the interaction between external and internal egg quality and may allow the development of a quality grading system for eggs based on non-destructive methods. This work represents one of the first studies in the field, as scarce literature addressing the association between internal and external egg quality is found in turkeys. In addition, the results of the present work will contribute to the limited knowledge of egg quality in an ‘alternative’ poultry species, as well as to the local breeds and backyard systems, in which the majority of the production of edible eggs from these species is actually produced. The results of this study will help breeders to enhance the consumption and value of their eggs and to develop marketing channels for these high-quality products.

## Material and methods

### Experimental unit

The sample that constituted the present study was 197 eggs laid by 22 adult Andalusian hens (70 weeks of age at the beginning of the study) from February 2019 to April 2020. Birds belonged to the conservation nucleus of the breed, which is placed in the ‘Agropecuary Provincial Center of Diputación de Córdoba’ (37°54′50.9″N–4°42′40.4″W), in Andalusia (Southern Spain). The birds themselves were not directly involved in the experiment but served as the source of the analyzed eggs. Therefore, no special ethical approval was required from the Ethics Committee of Animal Experimentation of the University of Córdoba, as protocols of zootechnical purposes are exempted, as detailed in the Spanish (Royal Decree Law 53/2013) and European legislation (European Union Directive 2010/63/UE dated 22 September 2010). Animals were kept in outdoor dirt pens during the whole experiment, with free access to a covered area. This represents the actual conditions under which this genotype is reared and simulates the organic-like housing standards. Animals had ad libitum access to feed as well as to fresh and clean water during the extension of the collection period. Animals were fed during the whole experiment with the same commercial feed, whose composition is detailed in Table 1. In addition to the commercial feed, since animals were placed in outdoor pens, they had access to other foods such as pasture and insects, when available.

**Table 1:** Ingredients and nutrient composition of the commercial feed.

Recipe		
Corn, shelled toasted soybeans flour, barley, coarse calcium carbonate, wheat, calcium carbonate, soybean oil, monocalcium phosphate, sodium chloride, sodium bicarbonate		
**Analysed nutrient composition (%)**	Crude protein	15.70
	Crude lipids	3.60
	Crude fiber	2.40
	Crude ash	14.00
	Calcium	4.10
	Phosphorus	0.66
	Sodium	0.15
	Methionine	0.38
	Lysine	0.79

Egg collection and analysis occurred every 14 days at first time of the morning. Collected eggs were measured within the 24 hours after oviposition at room temperature in the facilities of the Agropecuary Provincial Centre. From collection to analysis, eggs were individually identified and stored in room temperature conditions. The external quality traits measured were weight (g) and shape index. This index was obtained from the major (ØM) and minor (Øm) diameter of the intact shell, according to the following formula:

$$SI=\;\frac{Øm}{ØM}\times 100$$

Internal quality measures comprised the percentage of each component (eggshell, albumen, and yolk) on the total weight of the egg, albumen height, yolk diameter, yolk colorimetry (lightness ‘L*’, green-red axis ‘a*’, and blue-yellow axis ‘b*’), DSM® fan (formerly known as Roche yolk color fan) and both albumen and yolk pH. The percentage of the egg’s components was estimated from the weight of each component compared to the total weight of the egg. Yolk colorimetry was determined using a portable spectrophotometer (CM 700d, Konica Minolta Holdings Inc., Tokyo, Japan), and results were expressed using the International Commission on Illumination (CIE) L*a*b* system, according to the bibliography (González Ariza et al., 2022a, 2019).

### Data analysis

Eggs were classified according to their shape index and weight. Shape index clusters were those developed in the commercial egg market, identifying sharp (SI < 72), standard (72 ≤ SI ≤ 76), and rounded eggs (SI > 76). On the other hand, as there are no commercial standards for turkey egg weight, three weight groups were made according to sample terciles, differentiating light (74.54 – 77.31 g), medium (77.33 – 85.69 g) and heavy (85.77 – 100.06 g) eggs. Seven categories were obtained when combining the clusters of shape index and weight of the eggs: ‘Sharp-Light’, ‘Sharp-Medium’, ‘Sharp-Heavy’, ‘Standard-Light’, ‘Standard-Medium’, ‘Standard-Heavy’ and ‘Rounded-Light’ (Table 2). The groups ‘Rounded-Medium’ and ‘Rounded-Heavy’ were removed due to sample size problems, as only 2 and 1 observations were obtained per group, respectively.

**Table 2:** Egg categories obtained from the combination of weight and shape index groups.

		Weight		
		74.54 – 77.31 *g*	77.33 – 85.69 *g*	85.77 – 100.06 *g*
**Shape Index**	**< 72**	Sharp – Light	Sharp – Medium	Sharp – Heavy
	**72 – 76**	Standard – Light	Standard – Medium	Standard – Heavy
	**> 76**	Rounded – Light	–	–

A DCA was performed to determine the extent to which the variance of internal quality attributes explained the categories created by the external quality attributes. For this, egg shape and weight clusters were considered dependent variables, while the aforementioned internal quality traits were considered independent variables. The analysis was performed by the Discriminant Analysis routine of the Analyzing Data package of XLSTAT Pearson Edition.

### Preliminary multicollinearity test

Multicollinearity represents the degree of linear correlation between the explanatory variables included in an analysis (González Ariza et al., 2022a). In other words, multicollinearity depicts the proportion of the explanatory variance of a variable that is already explained by other variables or their combination. Thus, as they do not contribute to additional variance and overinflate the explicative properties of the model, redundant variables must be detected and removed from further analysis.

Among different methods employed to measure multicollinearity, the variance inflation factor (VIF) and tolerance have been successfully employed in meat and egg quality studies (González Ariza et al., 2021, 2022a, 2022b; Salgado Pardo et al., 2024, 2023). It is based on the measure of the ratio of variance in a regression model with multiple attributes divided by the variance of a single-attribute model (James et al., 2013). Multicollinearity is detected when *k* vectors lie in a subspace of dimension less than *k*. VIF was computed as a subroutine of the Discriminant Canonical Analysis routine of the Analyzing Data package of XLSTAT software (Addinsof Pearson Edition 2014, Addinsof, Paris, France).

$$VIF=\;\frac{1}{1-R^{2}}$$

Were R^2^ is the coefficient of determination of the regression equation. A maximum VIF value of 5 was considered, according to literature (González Ariza et al., 2021, 2022b).

### Discriminant canonical analysis efficiency and model reliability

Wilk’s lambda test is commonly employed to identify those variables significantly contributing to the discriminant function. According to this technique, when values tend to 0, the greater the contribution of the variable to the function. Discriminant functions are employed to explain the adscription of clusters when the significance (tested using χ^2^) is under 0.05 (González Ariza et al., 2021).

Due to the unequal nature of sample sizes, Pillai’s trace criterion was employed to assess model reliability through the assumption of equal covariance matrices. This was performed through the subroutine of the Discriminant Canonical Analysis routine of the Analyzing Data package of XLSTAT software (Addinsof Pearson Edition 2014, Addinsof, Paris, France). Obtaining a significance value under 0.05 is indicative of statistical significance and, therefore, allows the application of the DCA (González Ariza et al., 2022b).

### Variable dimension reduction

The preliminary component analysis (PCA) allows for reducing variables by removing those less significant in the model. Thus, a preliminary PCA was implemented using the Discriminant Analysis routine of the XLSTAT software.

### Canonical coefficients and loading interpretation

The use of DCA allows the description of the correct assignment rate of eggs within their category (shape-weight groups). Variables obtaining a discriminant loading ≥ |0.40| were considered substantially discriminant, and non-significative variables were excluded. To this respect, the greater the absolute coefficient of a variable, the greater the explicative ability (González Ariza et al., 2021). Data were standardized and squared Mahalanobis distances were calculated according to the following formula:

$$D_{ij}^{2}=\left(\bar{Y_{i}}-\;\bar{Y_{j}}\right)\;COV^{-1}\left(\bar{Y_{i}}-\;\bar{Y_{j}}\right)$$

where D^2^_ij_ represents the distance between population i and j; Ῡ_i_ and Ῡ_j_; are the means of variable x in the i^th^ and j^th^ populations, respectively and COV^−1^ is the inverse of the covariance matrix of measured variable x.

Squared Mahalanobis distances were employed to graphically represent the clustering patterns of egg categories attending to the differences in their internal quality attributes. To this goal, a dendrogram with the egg categories was built up using the underweighted pair-group method arithmetic averages (UPGMA) from the Universität Rovira i Virgili (URV), Tarragona, Spain, and the Phylogeny procedure of MEGA X 10.0.5 (Institute of Molecular Evolutionary Genetics, The Pennsylvania State University, State College, PA, USA).

### Cross-validation of the discriminant function

A hit ratio was computed through the percentage of accurate assignments of observations within their actual category. To this respect, the leave-one-out cross-validation was implemented to validate the discriminant functions used, according to literature (González Ariza et al., 2022a; Salgado Pardo et al., 2024). To consider a classification rate accurate enough, it must be at least 25 % higher than obtained by chance (González Ariza et al., 2021, 2022b). Press’ Q significance test was used to compare the discriminating power of the cross-validated function by using the following formula:

$$\text{Pres}s^{{}^{\prime }}Q=\frac{N-{\left(\text{nK}\right)}^{2}}{N\left(K-1\right)}$$

where N is the number of total observations; n is the number of observations correctly classified; and K is the number of categories. Press’ Q value was compared to the critical value of 6.63 for χ^2^ considering one degree of freedom and a significance of 0.01. Then, if the value of Press’ Q statistic was under χ^2^ = 6.63, the cross-validated classification could be considered significantly better than chance.

## Results

### Preliminary multicollinearity test and DCA model reliability

The preliminary multicollinearity test reported yolk percentage as the only redundant variable among the traits included in the analysis (VIF > 5), and hence, was discarded for further analysis. A summary of the VIF and tolerance of the rest of the variables is shown in Table 3.

**Table 3:** Results of the preliminary multicollinearity analysis of egg’s internal quality attributes.

Statistics/Variables	Tolerance	VIF
Yolk a*	0.365	2.738
Yolk b*	0.430	2.326
DSM® fan	0.580	1.725
% Shell	0.655	1.527
Yolk L*	0.715	1.398
% Albumen	0.744	1.344
Albumen height	0.747	1.338
Albumen pH	0.757	1.321
Yolk diameter	0.813	1.230

VIF = 1 (not correlated); 1 < VIF < 5 (moderately correlated); VIF ≥ 5 (highly correlated).

As found in Table 4, Pillai’s trace criterion reported significant differences across the internal quality attributes of egg weight and shape categories (*p*< 0.05), which enabled the performance of the DCA.

**Table 4:** Results of Pillai’s trace of equality of covariance matrices of canonical discriminant functions to test the suitability of data for a discriminant canonical analysis.

Parameter	Value
Pillai’s Trace Criterion	0.909
F (Observed value)	3.268
F (Critical value)	1.331
df1	60
df2	1098
Significance	<0.0001
alpha	0.05

F, Snedecor’s F; df1, numerator degrees of freedom for the F-approximation; df2, denominator degrees of freedom for the F-approximation.

### Canonical coefficients, loading interpretation, and spatial representation

Once redundant variables were discarded, the test of equality of group means across the categories of egg shape and weight described five statistically significant variables. On the other hand, albumen percentage (%), yolk pH, yolk redness (a*), yolk yellowness (b*), and DSM® fan reported non-significant results in the discrimination function (*p*> 0.05). Significant variables were ranked in increasing order for F (Snedecor’s F) and decreasing Wilk’s Lambda value (Table 5). For a significant variable, higher values for F and lower values for Wilk’s Lambda correspond to a higher degree of discrimination. Furthermore, means values of internal quality traits by egg shape and weight categories are shown in Table 6.

**Table 5:** Results for the tests of equality of group means across egg shape-weight categories.

Variables	Rank	Wilks’ Lambda	F	df1	df2	Significance
Yolk diameter	1	0.483	33.402	6	187	<0.0001
% Shell	2	0.730	11.528	6	187	<0.0001
Albumen height	3	0.893	3.718	6	187	0.002
Yolk L*	4	0.918	2.796	6	187	0.013
Albumen pH	5	0.921	2.681	6	187	0.016

F, Snedecor’s F; df1, numerator degrees of freedom for the F-approximation (groups minus 1); df2, denominator degrees of freedom for the F-approximation (observations minus 1).

**Table 6:** Means of eggs’ internal quality traits by weight and shape index classes.

Shape-weight category	Sharp-Heavy	Standard-Heavy	Sharp-Medium	Standard-Medium	Sharp- Light	Standard - Light	Rounded - Light
Albumen height (mm)	8.449	8.222	9.476	9.919	9.315	9.723	10.136
Roche yolk fan	10.471	10.548	10.800	10.462	10.583	10.647	11.333
Yolk L*	48.751	49.909	47.386	49.227	46.958	47.899	46.796
Yolk a*	4.590	4.369	4.552	5.120	4.537	5.064	5.854
Yolk b*	24.063	27.646	24.404	27.135	21.639	22.075	23.553
Yolk diameter (mm)	50.630	49.484	45.482	46.607	43.613	44.285	45.411
Shell (%)	11.262	12.026	13.209	12.989	13.889	14.062	13.725
Albumen (%)	50.212	53.532	53.128	56.307	55.426	57.570	52.451
Yolk pH	6.211	6.229	6.177	6.308	6.229	6.162	6.289
Albumen pH	8.436	8.564	8.281	8.236	8.433	8.311	8.208

**Fig. 2:**
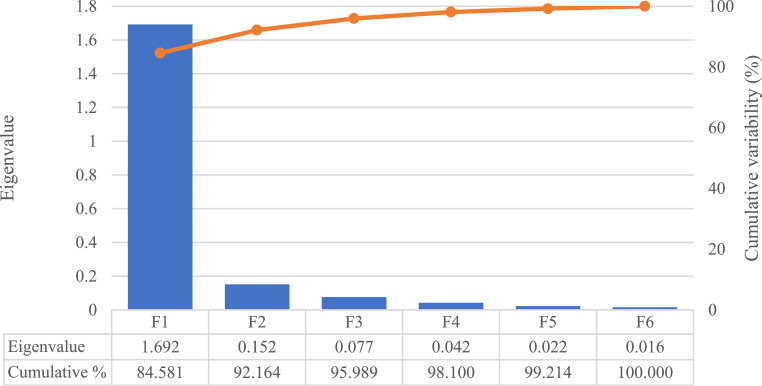
Percentage of self-explained and cumulative variance of each canonical variable function (F).

**Fig. 3:**
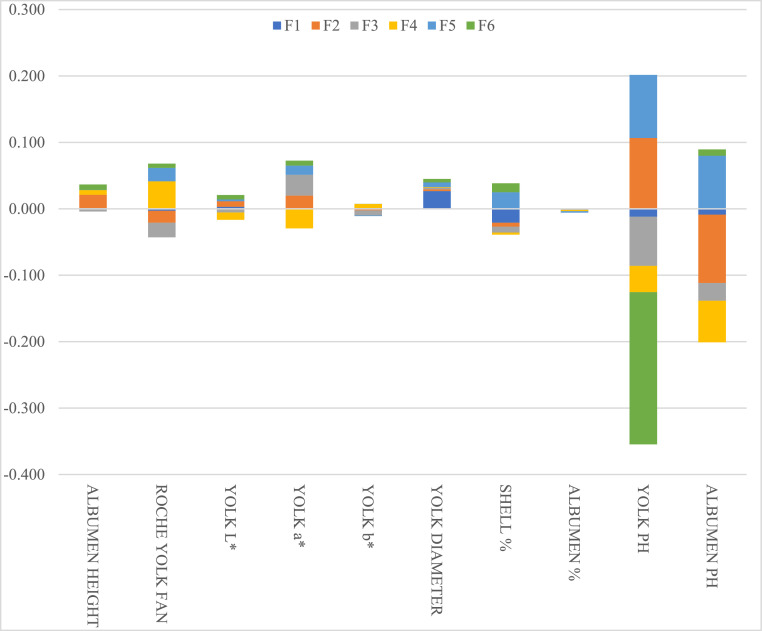
Discriminant loadings for egg internal quality attributes determining their importance on each canonical discriminant function (F).

From a total of six discriminant functions described by the model, the first function (F1) showed a discriminatory power of 84,58 % of the variance by itself (Fig. 2). The relative importance (loading) of each egg internal quality trait across the six discriminant functions is depicted in Fig. 3.

**Fig. 4:**
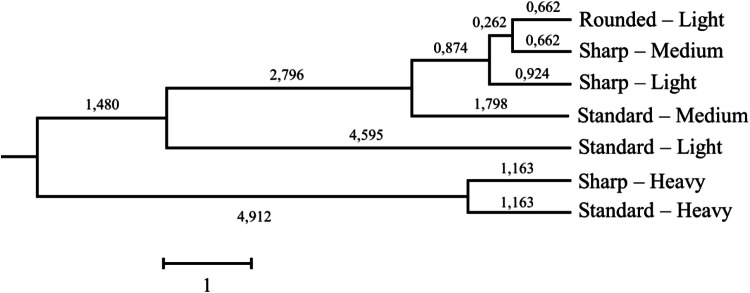
Dendrogram depicting the Mahalanobis distances between eggs’ weight and shape index categories.

A dendrogram representing the Mahalanobis distances of the different egg categories is shown in Fig. 4. It was built through the translation of the relative distance of a problem egg observation to the closest egg category through its internal quality attributes. Hence, the likelihood of observation matching the categories was estimated as previously described in the literature (Salgado Pardo et al., 2023).

### Result of the cross-validation test and reliability of the model

The cross-validation test was performed and classification and leave-one-out matrices were obtained (Table 7). The model presented good discriminating properties, as obtained a 95,88 % of correct assignations of observations to their respective groups. The only egg categories not showing 100 % of correct assignments were ‘Sharp – Light’ and ‘Standard – Medium’. Observations from the ‘Sharp – Light’ category (88,89 % of correct assignments) were confounded with the other two light categories; ‘Standard – Light’ (8,33 %) and ‘Sharp – Light’ (2,78 %). On the other hand, ‘Standard – Medium’ observations (89,74 % of correct matching rate) were misclassified as ‘Round – Light’ (5,23 %), ‘Standard – Heavy’ (2,56 %) and ‘Standard – Light’ (2,56 %).

**Table 7:** Results of the leave-one-out cross-validation confusion matrix (%) of correct assignments of observations within their category.

	Round - Light	Sharp - Heavy	Sharp -Med.	Sharp - Light	Stand. - Heavy	Stand. - Med.	Stand. - Light	Total	% correct
Round - Light	12	0	0	0	0	0	0	12	100.00 %
Sharp - Heavy	0	34	0	0	0	0	0	34	100.00 %
Sharp - Med.	0	0	25	0	0	0	0	25	100.00 %
Sharp - Light	1	0	0	32	0	0	3	36	88.89 %
Stand. - Heavy	0	0	0	0	31	0	0	31	100.00 %
Stand. - Med.	2	0	0	0	1	35	1	39	89.74 %
Stand. - Light	0	0	0	0	0	0	17	17	100.00 %
Total	15	34	25	32	32	35	21	194	95.88 %

Where ‘Stand.’ means ‘Standard’ and ‘Med.’ Means ‘Medium’.

## DISCUSSION

The diversification of production systems is an essential requirement for the long-term and sustainable development of farming and animal protein supply. The combination of different species allows exploiting their strengths through their respective feeding, behavioral, and adaptive peculiarities to maximize production efficiency. Furthermore, diversifying not only species but also breeds has a positive impact on overall diversity (Bondoc et al., 2020). Therefore, the results of the present work provide new knowledge in the undeveloped field of turkey egg quality, as well as contribute to the scarce literature addressing indigenous genotypes, which is not always a priority in research efforts.

Concerning the insufficient sample size in the ‘Round – Standard’ and ‘Round – Heavy’ categories, the lack of suitability of the shape standards of the commercial egg industry could be suggested for the turkey species. The range of shape indexes described in the present study varied from 61.38 to 78.29, with a mean value of 72.09, which is almost the limit for eggs to be considered ‘sharp’ (SI < 72) according to these standards (González Ariza et al., 2022a). Therefore, although significant differences were obtained in the present study using this classification criterion, the absence of these ‘round’ groups (SI > 76) highlights the need to develop a specific shape standard for turkeys.

The preliminary test for multicollinearity showed that the yolk percentage was the only redundant variable. In this regard, the literature has widely described the positive correlation between yolk weight with yolk diameter (Abacı et al., 2023; Kul and Seker, 2004; Yahaya et al., 2021), shell weight (Abacı et al., 2023; Portillo-Salgado et al., 2020; Sezai et al., 2013), and albumen weight and height (Abacı et al., 2023; Kul and Seker, 2004). Therefore, the finding of multicollinearity in yolk percentage does not mean that the proportion of yolk remains unchanged with variations in egg weight and shape, but that its contribution to the variance explanation is already covered by other variables, possibly yolk diameter or the percentage of shell and albumen.

In fact, yolk diameter was the internal quality trait with the greatest explanatory potential among egg categories. This is consistent with the literature on almost all poultry species, which reports that yolk diameter is closely related to weight (Abacı et al., 2023; Begli et al., 2010; Kul and Seker, 2004; Oblakova, 2006; Portillo-Salgado et al., 2020; Sarica et al., 2012; Sezai et al., 2013) and shape index (Abacı et al., 2023; Begli et al., 2010; Portillo-Salgado et al., 2020; Sarica et al., 2012; Sezai et al., 2013). This close association between egg weight and shape with yolk size could be explained by the process of egg formation. The yolk enters the oviduct during ovulation and its movement stimulates the deposition of egg white proteins (Nys and Guyot, 2011). Different yolk sizes would therefore result in a greater or lesser stimulation of the mucosa, and therefore, a different deposition of albumen, which accounts for 53.8 % of the total egg weight (Hasan and Khaliduzzaman, 2022). A similar mechanism has been reported for mineral deposition (Nys and Guyot, 2011), but this is a parameter in which many other factors are involved. Therefore, the most common assumption is that yolk size increases egg weight by increasing the weight of the other components, as it is positively correlated with albumen (Abacı et al., 2023; Kul and Seker, 2004; Oblakova, 2006; Sarica et al., 2012) and shell weight (Abacı et al., 2023; Hristakieva et al., 2017; Kul and Seker, 2004; Oblakova, 2006; Portillo-Salgado et al., 2020; Sarica et al., 2012; Sezai et al., 2013). This same trend is found in the present results, as the mean by classes, as heavier eggs had wider yolk diameters and vice versa.

On the other hand, the association between shape index and yolk width is more complex and controversial. Egg shape is mainly determined during the formation of the shell membranes, which takes place in the isthmus (Duan et al., 2016). In addition, the hydration process of the developing egg, which takes place in the distal part of the magnum (Nys and Guyot, 2011) causes the dilatation of the structure, and its contact with the uterine walls would also contribute to its ovoid shape (Nys and Guyot, 2011). Nevertheless, there is not a clear influence of yolk size in the aforementioned process, but there is for the deposition of yolk-associated structures. In this regard, increasing yolk size has been proposed to lead to greater deposition of denser albumen and surrounding membranes, which occurs in the equatorial axis of the egg (Duman et al., 2016). Thus, larger yolks would be associated with wider and rounder eggs. This is consistent with the present results since the yolk diameter increased with the shape index within the medium and light categories. Particularly striking was the case of the ‘Round – Light’ cluster, which achieved yolk sizes close to those of heavier eggs. However, contradictory results have been reported in the literature for different poultry species. While a positive correlation between yolk size and shape index has been described in Guinea fowl (Sezai et al., 2013), on the other hand, turkeys (Oblakova, 2006; Portillo-Salgado et al., 2020) and Japanese quails (Kul and Seker, 2004) have reported negative correlations. In chickens, positive and negative correlations as well as no correlation have been reported (Abacı et al., 2023; Begli et al., 2010; Duman et al., 2016; Sarica et al., 2012). This may suggest that the correlation between yolk size and egg shape is highly influenced by the species and genotype. Finally, the negative trend of yolk diameter on the shape index described for the ‘heavy’ categories could suggest that a sufficiently large structure size could reduce the effect of the yolk on egg shape. Moreover, this could also be a consequence of the lack of suitability of commercial chicken shape categories for turkeys, once again.

The second most discriminant variable was the percentage of shell in the total egg weight. Attending to the means by categories, proportionally heavier shells were found in light eggs, whose percentage reduced with increasing egg weight. The inverse relationship between egg weight and shell percentage has been reported in turkeys (Javid et al., 2017; Mróz et al., 2014; Taskin et al., 2016), quail (Kul and Seker, 2004) and chicken (Iqbal et al., 2017), whereas a positive correlation was reported for Guinea fowl (Sezai et al., 2013). On the other hand, no clear association is observed between shell percentage and shape index, which is in line with the inconsistency found in the bibliography. Light, non-significant positive associations have been reported in turkeys (Hristakieva et al., 2017; Oblakova, 2006), Guinea fowl (Sezai et al., 2013) and chicken (Begli et al., 2010; Duman et al., 2016; Sarica et al., 2012). Therefore, shell percentage variation has been suggested to be more dependent on overall weight than shape index.

The third most discriminating variable in the analysis was albumen height. The close relationship between this variable with other quality traits (Abacı et al., 2023; Duman et al., 2016; Sarica et al., 2012) has led to albumen height being proposed as a selection criterion for egg quality (Begli et al., 2010). Moreover, this trait has been commonly employed to determine the quality of dense albumen (Kul and Seker, 2004; Sezai et al., 2013). In the present work, the thinnest albumens were reported by the ‘heavy’ categories. This could be attached to a lower concentration of dense proteins in the albumen as the weight of the egg increases and the albumen is responsible for more than half of the total weight (Hasan and Khaliduzzaman, 2022). The ‘Rounded – Light’ category exhibited the tallest albumen, followed by the ‘standard’ and, eventually, ‘sharp’ categories, with independence of their weights. This strong influence of shape on albumen height could be because dense albumen is deposited along the equatorial axis of the egg (Duman et al., 2016) to prevent yolk’s displacements. Therefore, greater deposition of dense albumen could result in a more rounded egg shape. There are no similar reports in the literature, where no clear correlation has been reported in turkeys (Oblakova, 2006), while the inverse correlation has been reported in Japanese quail (Kul and Seker, 2004), Guinea fowl (Sezai et al., 2013) and chickens (Abacı et al., 2023; Begli et al., 2010).

The fourth discriminating variable in the rank was the yolk lightness axis (L*). In this respect, the ‘heavy’ categories tended to descriptively present lighter yolk means, followed by medium and, closely to them, light eggs. On the other hand, standard eggs seem to present descriptively and slightly lighter yolks than sharp and round eggs. Due to the similarities observed in the trends of the category means between this variable and yolk diameter, eggs increasing in weight could be suggested to present larger and lighter yolks, possibly due to a lower pigment concentration. Among the scarce literature reporting similar results, egg weight in chicken has been reported to increase yolk lightness (Şekeroǧlu and Altuntaş, 2009) and to reduce the overall proportion of orange color (González Ariza et al., 2022a). In terms of shape, a slight correlation has been proposed between the width and length of the shell with the dullness and yellowish of the yolk in chicken (González Ariza et al., 2019). On the other hand, a slight negative association (Alipanah et al., 2013) and no association between traits have been described in other poultry species (Duman et al., 2016; Sarica et al., 2012). The limited nature of reports may in part be a consequence of the general lack of inclusion of yolk colorimetry observed in papers addressing the correlation between internal and external egg quality.

Albumen pH was the last variable in the rank order reporting explanatory properties. This variable is commonly employed to determine egg freshness, due to its ability to reflect storage periods (González Ariza et al., 2021). Since the eggshell has a porous nature, gas exchange occurs during storage, and losses of CO_2_ cause the alkalinization of the albumen (Heath, 1977). In the present work, as all eggs were stored for the same period and exposed to the same environmental conditions, no effect of storage is expected across categories. Therefore, these differences in pH could be the consequence of a different gas exchange activity across sizes and shapes. To this respect, larger eggs present a greater surface area, and hence, could suffer from an increased gas exchange activity compared to smaller eggs (Baykalir and Aslan, 2020). This is in line with the results of the present work, as slightly higher pH values were obtained in heavier eggs. On the other hand, no clear associations between gas exchange and shape index are found in the bibliography, possibly due to the use of two different formulae to compute the egg’s surface area. While some authors refer to the formula based on the maximum length and width of the shell (Mohsenin, 1970), others refer to the formula based on the weight of the egg (Carter, 1975). Nevertheless, no clear effect of shape is visible when attending to the mean by class table.

Lastly, the results of the Mahalanobis distances reflect the strong clustering capacity of weight for the heavy categories, which were quite differentiated from the lighter categories. However, within medium and light categories, no clear effect of weight in the clustering pattern is found, but shape does, as standard and sharp eggs seem to be depicted separately. Finally, the model suggests the close similarities between the internal quality of “Rounded – Light” and “Sharp – Medium” eggs, as has been mentioned for traits such as yolk diameter, albumen pH, or shell percentage.

## Conclusion

This work provides the association between the internal and external quality of turkey eggs and allows knowing the internal quality of different commercial gradings. Furthermore, not only is it feasible to use egg weight and shape to explain internal quality, as evidenced by the sufficient significance of Pillai’s trace criterion, but the model showed a high accuracy in the cross-validation test. However, due to the small sample size of round eggs and the sharp mean values of the sample, the need to develop a specific grading system for the shape index of turkey eggs is emphasized. The description of multicollinearity in yolk percentage suggests that it is more appropriate to include yolk diameter in egg quality studies, which was supported by the finding that yolk diameter is the most discriminant variable. Among the discriminant variables, yolk lightness was generally less included in similar studies, highlighting the need to include yolk colorimetry in egg quality research. The results of the present work would help turkey breeders to structure their sales according to a scientific criterion, increasing the value of those eggs associated with high-quality standards. Thus, in these markets demanding larger yolks and a lower percentage of shells, heavier eggs would be the most valuable products, irrespective of their shape. On the other hand, in those markets that value yolk color, albumen height, and better storage conservation, smaller and rounder eggs would be its star product. Further studies involving different turkey genotypes and rearing systems should be developed to achieve a more comprehensive understanding of the relationship between external and internal physical quality of eggs.

## Funding

Our research was funded by 10.13039/501100002351FEDER project P20_00893 and, during the covering period of a predoctoral contract (FPU Fellowship), the Spanish Ministry of Science and Innovation.

## Ethical statement

All the authors involved in this study (José Ignacio Salgado Pardo, Antonio González Ariza, José Manuel León Jurado, Juan Vicente Delgado Bermejo, María Esperanza Camacho Vallejo) declare that the animals were not directly involved in the experiment but served as the source of the analyzed eggs. Therefore, no special ethical approval was required from the Ethics Committee of Animal Experimentation of the University of Córdoba, as protocols of zootechnical purposes are exempted, as detailed in the Spanish (Royal Decree Law 53/2013) and European legislation (European Union Directive 2010/63/UE dated 22 September 2010).

## CRediT authorship contribution statement

**José Ignacio Salgado Pardo:** Writing – review & editing, Writing – original draft, Supervision, Software, Resources, Methodology, Investigation, Formal analysis, Data curation, Conceptualization. **Antonio González Ariza:** Writing – review & editing, Writing – original draft, Visualization, Validation, Supervision, Software, Resources, Methodology, Investigation, Funding acquisition, Formal analysis, Data curation, Conceptualization. **José Manuel León Jurado:** Writing – review & editing, Resources, Funding acquisition. **Juan Vicente Delgado Bermejo:** Writing – review & editing, Visualization, Validation, Supervision, Software, Resources, Project administration, Funding acquisition. **María Esperanza Camacho Vallejo:** Writing – review & editing, Writing – original draft, Validation, Resources, Project administration, Funding acquisition.

## Declaration of competing interest

All the authors involved in this study (José Ignacio Salgado Pardo, Antonio González Ariza, José Manuel León Jurado, Juan Vicente Delgado Bermejo, María Esperanza Camacho Vallejo) declare no conflict of interest.
